# Human umbilical cord blood-stem cells direct macrophage polarization and block inflammasome activation to alleviate rheumatoid arthritis

**DOI:** 10.1038/cddis.2016.442

**Published:** 2016-12-22

**Authors:** Tae-Hoon Shin, Hyung-Sik Kim, Tae-Wook Kang, Byung-Chul Lee, Hwa-Yong Lee, Yoon-Jin Kim, Ji-Hee Shin, Yoojin Seo, Soon Won Choi, Seunghee Lee, Kichul Shin, Kwang-Won Seo, Kyung-Sun Kang

**Affiliations:** 1 Adult Stem Cell Research Center, College of Veterinary Medicine, Seoul National University, Seoul, Republic of Korea; 2 Research Institute for Veterinary Science, College of Veterinary Medicine, Seoul National University, Seoul, Republic of Korea; 3 Institute for Stem Cell and Regenerative Medicine in Kangstem Biotech, Biomedical Science Building, Seoul National University, Seoul, Republic of Korea; 4 Division of Rheumatology, Department of Internal Medicine, Seoul Metropolitan Government-Seoul National University Boramae Medical Center, Seoul, Republic of Korea

## Abstract

Rheumatoid arthritis (RA) is a long-lasting intractable autoimmune disorder, which has become a substantial public health problem. Despite widespread use of biologic drugs, there have been uncertainties in efficacy and long-term safety. Mesenchymal stem cells (MSCs) have been suggested as a promising alternative for the treatment of RA because of their immunomodulatory properties. However, the precise mechanisms of MSCs on RA-related immune cells are not fully elucidated. The aim of this study was to investigate the therapeutic potential of human umbilical cord blood-derived MSCs (hUCB-MSCs) as a new therapeutic strategy for patients with RA and to explore the mechanisms underlying hUCB-MSC-mediated immunomodulation. Mice with collagen-induced arthritis (CIA) were administered with hUCB-MSCs after the onset of disease, and therapeutic efficacy was assessed. Systemic delivery of hUCB-MSCs significantly ameliorated the severity of CIA to a similar extent observed in the etanercept-treated group. hUCB-MSCs exerted this therapeutic effect by regulating macrophage function. To verify the regulatory effects of hUCB-MSCs on macrophages, macrophages were co-cultured with hUCB-MSCs. The tumor necrosis factor (TNF)-*α*-mediated activation of cyclooxygenase-2 and TNF-stimulated gene/protein 6 in hUCB-MSCs polarized naive macrophages toward an M2 phenotype. In addition, hUCB-MSCs down-regulated the activation of nucleotide-binding domain and leucine-rich repeat pyrin 3 inflammasome via a paracrine loop of interleukin-1*β* signaling. These immune-balancing effects of hUCB-MSCs were reproducible in co-culture experiments using peripheral blood mononuclear cells from patients with active RA. hUCB-MSCs can simultaneously regulate multiple cytokine pathways in response to pro-inflammatory cytokines elevated in RA microenvironment, suggesting that treatment with hUCB-MSCs could be an attractive candidate for patients with treatment-refractory RA.

Rheumatoid arthritis (RA) is a chronic autoimmune disease accompanied by progressive synovitis, destructive arthropathy and systemic complications. The pathogenesis of RA is complicated, but the orchestrated interaction of abundant pro-inflammatory cytokines and cellular components is known to have an essential role in RA progression. Frequently, RA is characterized by the undesirable activation of T cells, which leads to the abnormal production of autoantibodies, known as rheumatoid factors (RFs), against normal immunoglobulins. Subsequently, autoantibody-activated macrophages produce inflammatory cytokines, which contribute to the intense inflammatory responses leading to tissue damage and clinical manifestations.^1, 2^ Therefore, current therapeutic strategies for the treatment of RA target these cytokines. As tumor necrosis factor-alpha (TNF-*α*) has a principal role in the pathogenesis of RA, anti-TNF-*α* biologic agents have brought marked clinical achievement in RA patients.^3^ Moreover, interleukin (IL)-1 and IL-6 blockades have been introduced because these cytokines are reported to be involved in the pathogenesis of RA.^4^ However, despite the widespread use of targeted therapies, up to 50% of patients with RA still fail to respond adequately. In addition, these approaches may carry long-term side effects, including serious infections and malignancies.^5, 6^ Therefore, there are clear unmet demands to develop safe and effective therapeutics without the potential risk of complications.

Cell-based therapies utilizing mesenchymal stem cells (MSCs) have been spotlighted as a promising tool for the treatment of a wide range of immune-related diseases, such as graft-*versus*-host disease, inflammatory bowel disease, multiple sclerosis, atopic dermatitis and RA.^7, 8, 9, 10^ These therapeutic trials are based on the immunoregulatory capabilities of MSCs. Importantly, several groups have reported active interactions between MSCs and various types of both innate and adaptive immune cells, such as T lymphocytes, B lymphocytes, dendritic cells (DCs) and natural killer (NK) cells.^11^ Direct cell-to-cell contact and paracrine action by soluble factors have been reported to be crucial for the immunomodulatory ability of MSCs.^15, 16^ Our previous studies revealed the anti-inflammatory effects of xenogeneic human umbilical cord blood-derived MSCs (hUCB-MSCs) in murine experimental colitis and atopic dermatitis.^10, 17^ However, the therapeutic efficacy and the mechanisms of action can be altered by the disease-related immunologic microenvironment or the manipulation of MSCs.

Although several groups have demonstrated the different mechanisms of action for the preventive and curative efficacy of MSCs in RA, these groups have primarily focused on the regulation of immunocompetent cells, mainly autoreactive T and B lymphocytes.^18, 19^ More recently, accumulating evidence has shown that macrophages are responsible for the exacerbation of inflammatory responses and collateral damage in RA.^20^ Indeed, macrophages produce the core cytokines involved in RA pathogenesis, including TNF-*α* and IL-1*β*,^21^ which are targeted by current biologic medications. However, the underlying mechanisms by which MSCs regulate macrophage activation are relatively less well understood from the perspective of systemic immune homeostasis in response to the RA-related inflammatory microenvironment.

The inflammasome is a novel IL-1*β*-generating innate immune apparatus mainly located in monocytic cells. In particular, nucleotide-binding domain and leucine-rich repeat pyrin 3 (NLRP3) inflammasomes, the most well-established inflammasome, were recently reported to have an epistatic relation with RA.^22^ Namely, genetic mutations in the NLRP3 protein are closely associated with the susceptibility to and severity of RA.^23^ Moreover, NLRP3 inflammasome activity is enhanced in patients with RA.^24^ Therefore, targeting NLRP3 inflammasomes or their downstream pathways can be an effective strategy for attenuating RA.

The aim of this study was to investigate the therapeutic efficacy of systemically delivered hUCB-MSCs in a murine model of collagen-induced arthritis (CIA). To verify the mechanism responsible for their anti-inflammatory effects, we explored the possible mechanisms through which hUCB-MSCs may modulate multiple macrophage responses in terms of homeostatic immune balancing.

## Results

### Systemic delivery of hUCB-MSCs exerts therapeutic effect against mouse CIA model

**Figure 1:**
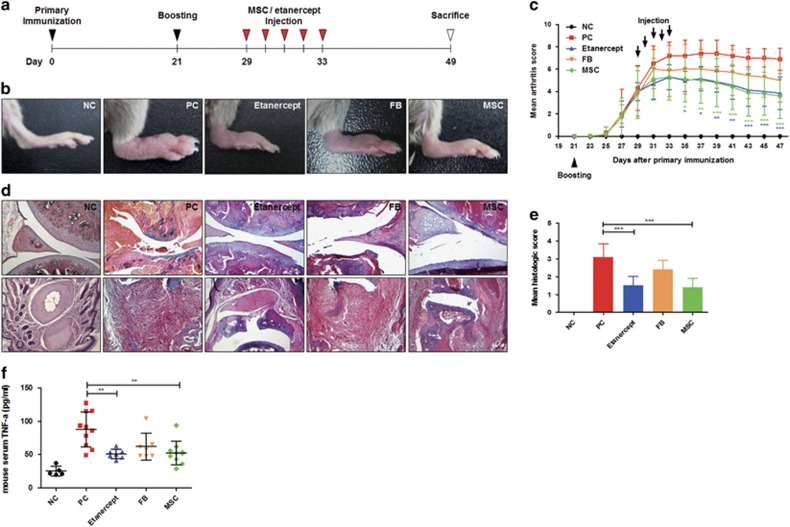
Intraperitoneal injection of hUCB-MSCs markedly ameliorates deterioration of experimental arthritis. (**a**) Schematic illustrating the protocol for CIA induction and hUCB-MSCs treatment. (**b**) Representative gross lesions of the hind limb were photographed for clinical assessment. (**c**) Clinical severity was consistently monitored, and arthritis score was calculated until sacrifice. *P<0.05, **P<0.01, ***P<0.001 *versus* PC (two-way ANOVA for the comparison of each time point). (**d** and **e**) All mice were sacrificed on day 49 for histopathological evaluation. Paraffin-embedded sections of both patellar and hind phalangeal joints were stained with H&E. Representative microscopic images of both joints are shown (**d**); histopathological integrity was calculated based on these images (**e**), scale bar=100 *μ*m. ***P<0.001 *versus* PC (one-way ANOVA followed by the Bonferroni *post hoc* test). (**f**) Serum TNF-*α* concentrations were measured with an ELISA at day 49. **P<0.01 *versus* PC (one-way ANOVA followed by the Bonferroni *post hoc* test). At least five mice per group were used: NC=negative control (black; *n*=5 mice), PC=positive control, untreated group (red; *n*=10 mice), etanercept (blue; *n*=7 mice), FB (yellow; *n*=7 mice), hUCB-MSCs (green; *n*=10 mice) and all the results are shown as the mean±S.D.

We previously demonstrated the therapeutic effect of hUCB-MSCs against experimental colitis and atopic dermatitis and revealed the mechanisms underlying these effects.^10, 17^ In this study, we first investigated whether the therapeutic effects of hUCB-MSCs could be reproduced in the CIA model of autoimmune arthritis, which shares many immunologic, histologic and clinical similarities with RA.^25^ We also investigated whether the route and frequency of administration could alter the extent of these effects. Multiple intraperitoneal injections of hUCB-MSCs greatly ameliorated the clinical severity of CIA, in contrast with the results observed in untreated and fibroblast (FB)-injected mice. Interestingly, hUCB-MSCs showed a therapeutic effect similar to that of etanercept, a TNF-*α* antagonist (Figures 1b and c). Upon histologic evaluation, reduced synovitis and articular destruction were observed in hUCB-MSC- and etanercept-treated mice (Figures 1d and e). To verify the effect of hUCB-MSCs on the production of inflammatory cytokines closely associated with CIA pathogenesis, serum TNF-*α* levels were determined. The serum level of TNF-*α* was increased by CIA induction and remarkably decreased by treatment with hUCB-MSCs or etanercept, whereas the infusion of FB did not significantly suppress TNF-*α* secretion (Figure 1f).

**Figure 2:**
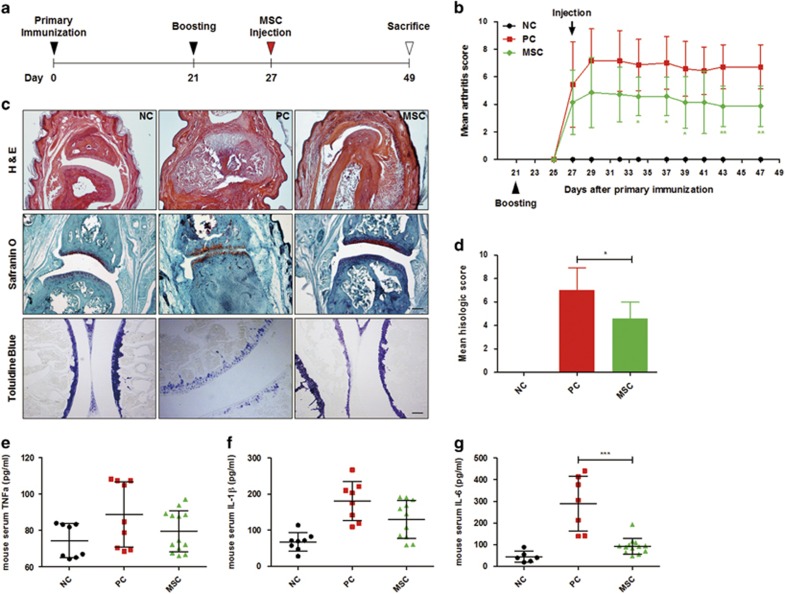
CIA is effectively attenuated by intravenous administration of hUCB-MSCs. (**a**) Outline of CIA induction and hUCB-MSC injection. Mice received a single intravenous injection of hUCB-MSCs after the onset of arthritis. (**b**) Clinical severity was evaluated every 2 or 3 days, and the clinical arthritis score was calculated until sacrifice (*n*=7 mice per group). *P<0.05, **P<0.01 *versus* PC (two-way ANOVA for the comparison of each time point). (**c** and **d**) After sacrifice, paraffin-embedded sections of joint tissue were stained with H&E, safranin O and toluidine blue for the evaluation of histologic articular damage and chondral destruction. Representative photomicrographs of hind interphalangeal joints stained with each dye are shown (**c**), scale bar=100 *μ*m, and histopathologic severity was assessed and calculated (**d**) (*n*=7 mice per group). *P<0.05 *versus* PC (one-way ANOVA followed by the Bonferroni *post hoc* test). (**e–g**) Serum levels of several pro-inflammatory cytokines, including TNF-*α* (**e**), IL-1*β* (**f**) and IL-6 (**g**), were determined by ELISA (*n*=6–13 mice per group). ***P<0.001 *versus* PC (one-way ANOVA followed by the Bonferroni *post hoc* test). All the results are shown as the mean±S.D.

Given that several studies have reported that a single intravenous injection of MSCs provides a protective and curative effect against CIA,^26^ we further confirmed the efficacy of a single injection of MSCs (Figure 2a). As expected, a single intravenous injection of hUCB-MSCs significantly attenuated the symptoms of arthritis (Figure 2b). Histologic damages, including pannus formation, synovitis and cartilage destruction, were markedly attenuated in the mice receiving hUCB-MSC administration compared with non-treated control mice (Figures 2c and d). Serum TNF-*α*, IL-1*β* and IL-6 levels were generally down-regulated by the infusion of hUCB-MSCs (Figures 2e–g). Among these inflammatory cytokines, the level of IL-6 decreased significantly. The injected cells were mostly distributed in the lung and joint tissue and were excreted within 2 weeks (Supplementary Figure S1). None of the mice treated with hUCB-MSCs showed any side effects or died until sacrifice.

Altogether, these findings demonstrate that the systemic administration of hUCB-MSCs can exert significant therapeutic effects against CIA without any noteworthy adverse effects.

### hUCB-MSCs suppress the activation of M1-type macrophages and induce the generation of M2-type macrophages via TNF-*α*-mediated activation of cyclooxygenase-2 (COX-2) and TNF-stimulated gene-6 (TSG-6)

**Figure 3:**
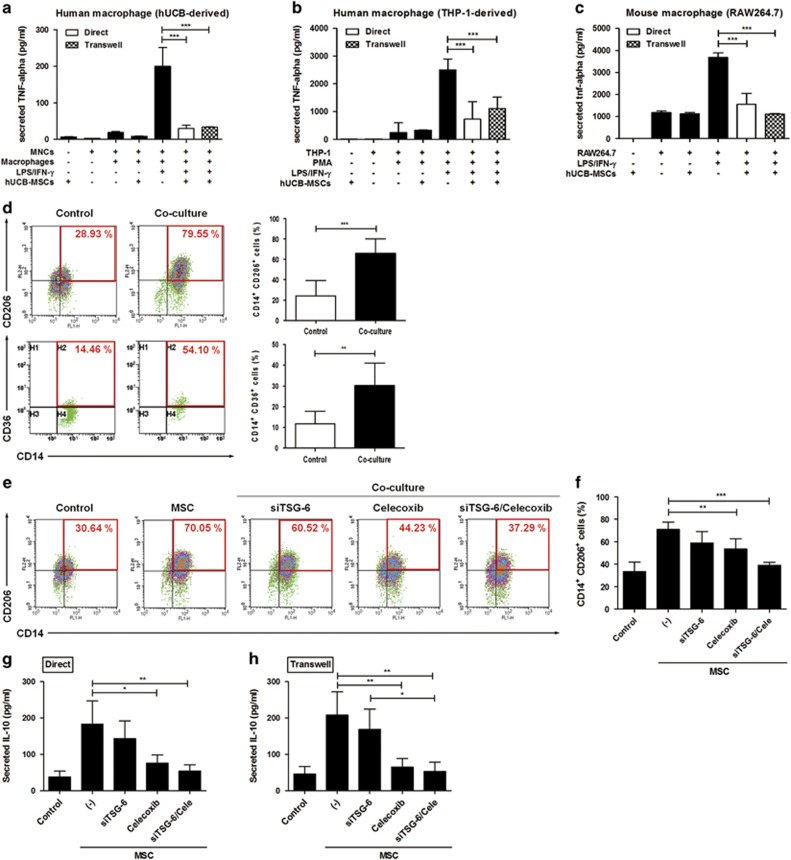
hUCB-MSCs suppress the activation of M1 macrophages and induce M2 polarization through the concerted action of COX-2 and TSG-6 signaling in response to TNF-*α*. (**a-c**) Human cord blood-derived (**a**) and THP-1-derived macrophages (**b**) or mouse macrophages (**c**) were activated with LPS and IFN-*γ* and subsequently co-cultured with hUCB-MSCs in a direct or transwell system. The TNF-*α* level was measured with an ELISA (*n*=at least three independent experiments). (**d**) Comparison of M2-specific surface marker expression on macrophages cultured alone (control) or co-cultured with hUCB-MSCs using flow cytometry. (**e** and **f**) hUCB-MSCs treated with a COX-2 and/or TSG-6 inhibitor were added to macrophages before analysis of alterations in CD206 expression in the CD14^+^ fraction (to determine M2 polarization) with flow cytometry. (**g** and **h**) The IL-10 level in the co-culture supernatants from direct (**g**) and transwell (**h**) condition was determined with an ELISA (*n*=3-4 independent experiments). Data shown are one representative or the cumulative of at least three independent experiments. All the results are shown as the mean±S.D. *P<0.05, **P<0.01, ***P<0.001 (one-way ANOVA followed by the Bonferroni *post hoc* test)

As cytokines such as TNF-*α* and IL-1*β*, which were down-regulated in the above study, are principally derived from macrophages, we sought to investigate whether hUCB-MSCs could modulate the phenotype or the function of macrophages *in vitro*.^1, 20, 27^ We thus generated macrophages from both cord blood-derived mononuclear cells (MNCs) and THP-1 cells. hUCB-MSCs were added to activated macrophages and co-cultured for 48 h. The secretion of TNF-*α* from M1 macrophages decreased significantly not only when cell-to-cell contact was allowed (Direct) but also when transwells were used (Transwell) (Figures 3a and c). Moreover, hUCB-MSCs converted macrophages into the anti-inflammatory M2 phenotype. The proportion of CD14^+^ cells expressing CD206, the well-established M2-type marker, was significantly higher in both the direct and transwell groups compared with the macrophage control group (Supplementary Figure S3A). As CD206 is known to be an unstable marker for M2 determination in THP-1-derived macrophages,^28^ the M2 polarization of THP-1-derived macrophages was confirmed by measuring the expression of CD36. Similar to the results for CD206^+^ cells in primary cultured macrophages, the proportion of CD36^+^ M2 macrophages was also increased by incubation with hUCB-MSCs (Figure 3d). These results indicate that hUCB-MSCs not only inhibit classical M1 activation but also elicit M2 polarization through a paracrine mechanism.

Considering that a large body of studies have demonstrated that immunomodulation by MSCs is not constitutive but is rather licensed by inflammatory cytokines,^29, 30, 31^ we hypothesized that the disease-specific inflammatory microenvironment may influence the regulatory effect of hUCB-MSCs. Therefore, the expression of crucial immunomodulatory factors in hUCB-MSCs was assessed after TNF-*α* pretreatment. COX-2 and TSG-6 were markedly increased (Supplementary Figure S3B). We thus inhibited these factors in hUCB-MSCs using selective inhibitors or small interfering RNA (siRNA). Interestingly, the M2 polarizing effect of hUCB-MSCs was almost entirely abrogated when COX-2 and TSG-6 were both inhibited, whereas individual inhibition of each factor only partially restored M2 polarization (Figures 3e and f). Consistently, the combined contribution of COX-2 and TSG-6 on M2 polarization was confirmed by determining the secretion of IL-10 (Figures 3g and h).

Taken together, our results suggest that hUCB-MSCs can regulate macrophage plasticity through the concerted action of COX-2 and TSG-6 signaling, which are enhanced in response to an RA-specific inflammatory milieu.

### hUCB-MSCs suppress the activation of the NLRP3 inflammasome in macrophages via an IL-1*β* feedback loop

**Figure 4:**
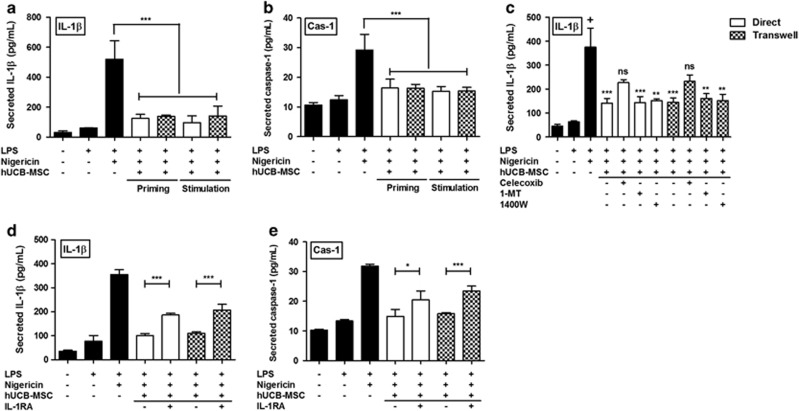
hUCB-MSCs negatively regulate NLRP3 inflammasome-mediated IL-1*β* and caspase-1 production in macrophages through an IL-1*β* feedback loop. (**a** and **b**) THP-1-derived macrophages were primed with LPS and subsequently stimulated with nigericin to activate the NLRP3 inflammasome. hUCB-MSCs were added to macrophages at either the LPS priming phase or the nigericin stimulating phase in direct or transwell conditions. Twenty hours later, the concentration of IL-1*β* (**a**) and caspase-1 (**b**) in the culture supernatant was measured with an ELISA. (**c**) hUCB-MSCs were added to LPS/nigericin-stimulated macrophages after pretreatment with 1-MT, 1400 W dihydrochloride and celecoxib. The IL-1*β* level was determined with an ELISA. (**d** and **e**) hUCB-MSCs were pretreated with an IL-1 receptor antagonist (IL-1RA) and co-cultured with LPS/nigericin-stimulated macrophages during nigericin stimulation. The level of IL-1*β* (**d**) and caspase-1 (**e**) in the supernatants was determined with an ELISA. All data are presented as the mean concentration±S.D. from three independent experiments. *P<0.05, **P<0.01, ***P<0.001; ns, not significant (one-way ANOVA followed by the Bonferroni *post hoc* test)

Along with TNF-*α*, IL-1*β* has been reported to have an essential role in RA pathogenesis.^1, 32^ Recently, increasing evidence has implicated the NLRP3 inflammasome-mediated IL-1*β* secretion in RA,^23^ and only a few groups have shown that MSCs could suppress the activation of NLRP3 inflammasomes in macrophages.^33, 34^ We thus investigated whether hUCB-MSCs could regulate the NLRP3 inflammasome. hUCB-MSCs were added to macrophages at either the lipopolysaccharide (LPS) priming step or stimulation step with nigericin. Adenosine triphosphate (ATP), another well-established NLRP3 activator, was also tested, but nigericin showed much higher efficiency than ATP (data not shown). The IL-1*β* and caspase-1 concentrations in the supernatant were significantly decreased by hUCB-MSCs, regardless of when the hUCB-MSCs were added (Figures 4a and b). As similar suppressive effects were observed at both hUCB-MSC addition time points, further experiments investigating the mechanism of inflammasome regulation were performed using the protocol adding hUCB-MSCs after the full activation of the NLRP3 inflammasome with nigericin. To explore the candidate factors mediating the suppressive effects of hUCB-MSCs on the NLRP3 inflammasome, we inhibited the synthesis or function of several well-known immunomodulatory factors using selective inhibitors. hUCB-MSCs were pretreated with 1-methyltryptophan (1-MT), 1400 W dihydrochloride or celecoxib for the blockade of indoleamine 2,3-dioxygenase (IDO), nitric oxide (NO) and COX-2, respectively. Interestingly, only COX-2 inhibition rescued the suppressive effect of hUCB-MSCs on inflammasome activation, whereas the blockade of IDO and NO did not show any significant changes (Figure 4c).

Consistent with previous findings, we hypothesized that the suppressive effect of hUCB-MSCs on NLRP3 inflammasome activation may be provoked by specific signals from macrophages. Therefore, we assessed the expression of crucial factors in hUCB-MSCs following incubation with or without the conditioned medium (CM) from macrophages after NLRP3 inflammasome activation. Interestingly, the CM from NLRP3 inflammasome-activated macrophages significantly up-regulated COX-2 expression in hUCB-MSCs compared with the CM from resting macrophages (Supplementary Figure S3C). Furthermore, COX-2 expression was enhanced when hUCB-MSCs were treated with recombinant human IL-1*β* (Supplementary Figure S3D), suggesting that NLRP3 inflammasome-mediated IL-1*β* production may contribute to the suppressive effect of hUCB-MSCs through a feedback mechanism. We thus inhibited IL-1*β* signaling in hUCB-MSCs with an IL-1 receptor antagonist (IL-1RA) to confirm the stimulatory effects of activated macrophage-derived IL-1*β* on hUCB-MSC function. As shown in Figures 4d and e, the inhibition of IL-1*β* signaling led to the partial loss in suppressive function of hUCB-MSCs on NLRP3 inflammasome in macrophages.

Taken together, our results indicate that hUCB-MSCs suppress the NLRP3 inflammasome in macrophages through an IL-1*β* feedback loop, suggesting that the therapeutic effect of hUCB-MSCs in RA may arise from the regulation of multiple macrophage functions by targeting various cytokines simultaneously.

### Immunologic profiling of serum and PBMCs from patients with RA

**Figure 5:**
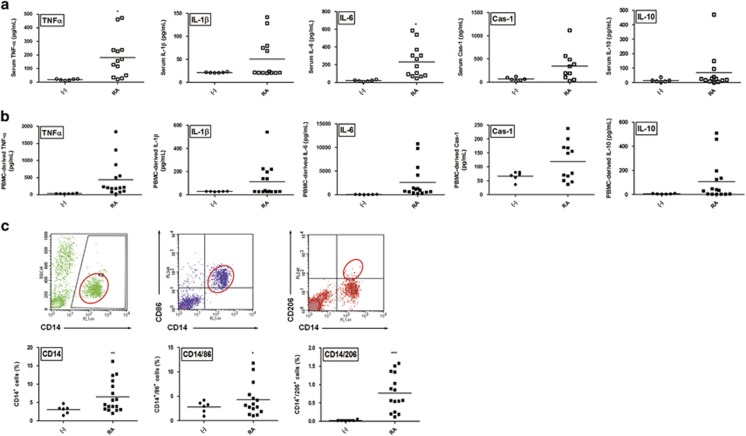
Baseline cytokine levels and the proportion of active immune cells are generally higher in RA patients. **(a** and **b)** The concentrations of major pro- and anti-inflammatory cytokines in the serum of patients (**a**) and cell culture supernatants from RA disease-related immune cells (**b**) were measured with an ELISA. Each point represents an individual and the mean concentration is shown as the line. (**c**) Representative dot plot of the monocyte/macrophage population and the M1/M2 macrophage subsets (red circles). A total of 15 samples from patients with active RA and six from healthy controls were used. The results are shown as the mean±S.D. *P<0.05, **P<0.01, ***P<0.001 (Student's *t*-test)

As hUCB-MSCs exerted regulatory effects on macrophages *in vitro*, we next investigated whether these effects could be reproduced *ex vivo* in cells from patients with active RA. To explore the systemic inflammatory status of patients with RA, we prepared serum and isolated peripheral blood mononuclear cells (PBMCs) from patients and healthy donors (Table 1). Subsequently, we analyzed the baseline concentrations of target cytokines. Although variations were observed among patients, the levels of TNF-*α*, IL-1*β*, IL-6 and even IL-10 were generally up-regulated in the serum, as well as in cell culture supernatants from patient PBMCs (Figures 5a and b). Furthermore, the PBMCs from patients with RA included a high proportion of both CD14^+^ CD86^+^ M1 and CD14^+^ CD206^+^ M2 macrophages compared with healthy controls (Figure 5c). These results show that diverse subsets of activated macrophages and the numerous cytokines released from these cells are elevated in the peripheral blood of patients, suggesting that macrophages may be intimately involved in systemic inflammation of RA.

### hUCB-MSCs consistently modulate immune cell functions beneficial for the amelioration of RA

**Figure 6:**
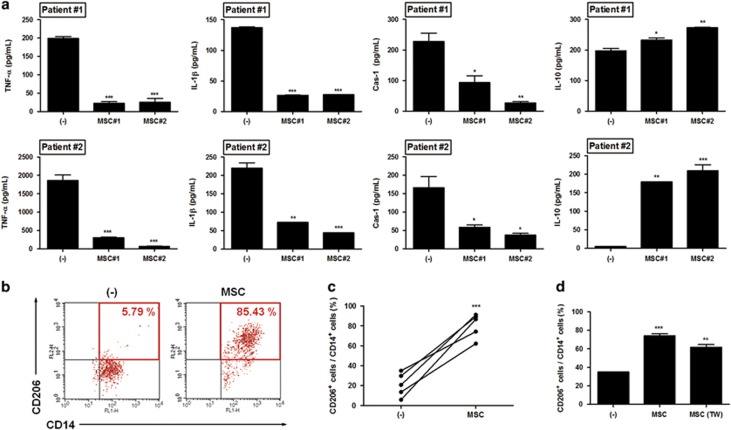
The excessive production of inflammatory cytokines by patient-derived PBMCs is remarkably suppressed by hUCB-MSCs. (**a**) The TNF-*α*, IL-1*β*, caspase-1 and IL-10 levels in the supernatants of patient-derived PBMCs co-cultured with hUCB-MSCs obtained from two different donors were measured using ELISA and compared with the levels secreted by PBMCs alone. (**b–d**) After co-culture with hUCB-MSCs in a direct or transwell system, the proportion of CD14^+^ CD206^+^ M2 cells in the PBMCs isolated from patients with RA or healthy controls was determined with flow cytometry. At least four freshly obtained peripheral blood samples were used in the patient and control groups. All data are shown as the mean±S.D. *P<0.05, **P<0.01, ***P<0.001; one-way ANOVA followed by the Bonferroni *post hoc* test (bar graph) and Student's *t*-test (line graph in **c**)

We next confirmed whether the regulatory effect of hUCB-MSCs is consistently reproducible in immune cells from RA patients. hUCB-MSCs were added to PBMCs and co-cultured for 2 days at MSC:PBMC ratios of 1 : 10 and 1 : 100. Consistent with previous *in vitro* results, the secretion of TNF-*α*, IL-1*β* and caspase-1 was significantly reduced by co-culture with two different donor-derived hUCB-MSCs, and IL-10 release was markedly up-regulated by hUCB-MSCs in the cells from a few patients (Figure 6a). Moreover, to explore alterations in macrophage plasticity, hUCB-MSCs were co-cultured with PBMCs for 2 days, after which cell surface markers were identified using flow cytometry. Surprisingly, hUCB-MSCs significantly increased the population of CD14^+^ CD206^+^ M2 macrophages in the PBMCs of all five patients examined (Figures 6b and d). Altogether, our results suggest that hUCB-MSCs can effectively regulate circulating macrophages in RA patients through the preferential induction toward M2 phenotype, as well as the inhibition of NLRP3 inflammasome-mediated IL-1*β* secretion, implying that these immune-balancing effects enable hUCB-MSCs to be a promising therapeutic option for RA treatment.

## Discussion

To date, a large body of studies has focused on demonstrating the therapeutic effects of MSCs in rheumatic diseases including RA; however, inconsistent results have been reported, with variations from no remarkable effects to significant amelioration. The systemic delivery of human adipose-derived MSCs was shown to exert significant therapeutic effects against CIA.^18, 35^ On the contrary, a few studies using allogeneic bone marrow-derived MSCs (BM-MSCs) have reported the opposite result.^36, 37^ These conflicting results may be attributed to several variables, including the origin of the MSCs, the number of cells injected and the route of administration. As we showed the anti-inflammatory effect of hUCB-MSCs in experimental colitis and atopic dermatitis,^10, 17^ we sought to investigate the therapeutic efficacy of hUCB-MSCs in CIA. In this study, we verified that the systemic administration of xenogeneic hUCB-MSCs can significantly ameliorate CIA, probably by re-establishing a homeostatic inflammatory milieu. Based on our evaluation of clinical symptoms, hUCB-MSCs appear to be as effective as etanercept, one of the most effective biologic drugs targeting TNF-*α*. Given that anti-TNF-*α* therapies provide clinical benefits for RA patients,^38^ these findings suggest that hUCB-MSCs can serve as a promising substitute for current therapeutics. Interestingly, the local intra-articular delivery of hUCB-MSCs showed little efficacy compared with systemic administration (data not shown), implying that systemic immune constitution rather than the reduction of local inflammation may be required for efficient therapy. The cell tracking experiment in our study showed that intravenously injected hUCB-MSCs migrated to the joint and lung tissue shortly after administration. Subsequently, the injected cells disappeared within 2 weeks, in agreement with previous reports showing the distribution of intravenously injected MSCs.^39^ Thus, in spite of the short period of cell distribution, it seems that systemically infused hUCB-MSCs can effectively suppress local and systemic inflammatory cascades.

MSCs have been reported to exert therapeutic potential in experimental arthritis by inhibiting Th1 and Th17 responses and inducing regulatory T-cell responses.^26, 40^ Recently, accumulating evidence has shown that macrophages have an essential role in the development of chronic inflammatory responses with direct tissue injury.^20, 41^ Therefore, we aimed to demonstrate the effects of MSCs on macrophages because the regulation of macrophages can be an attractive strategy for the treatment of RA. Previous studies have demonstrated that MSCs attenuate zymosan-induced mouse peritonitis by reducing pro-inflammatory cytokine production by macrophages.^42^ Moreover, MSCs have already been reported to induce the preferential conversion of macrophages to the M2 phenotype through soluble mediators such as prostaglandin-E_2_ (PGE_2_), IL-6, granulocyte-macrophage colony-stimulating factor and IL-1RA.^30, 43, 44, 45^ Consistent with these reports, in this study, hUCB-MSCs not only downregulated the secretion of TNF-*α* from activated macrophages but accelerated anti-inflammatory M2 polarization in a paracrine manner.

NLRP3 inflammasomes have been reported to be closely associated with RA susceptibility and responsiveness to anti-TNF-*α* biologic agents.^23, 24^ However, the mechanisms regulating NLRP3 inflammasome activation remain poorly understood. Oh *et al.*^33^ reported that hBM-MSCs negatively regulate the NLRP3 inflammasome in macrophages by decreasing mitochondrial reactive oxygen species (ROS), with this inhibitory effect primarily being mediated by stanniocalcin-1. More recently, several studies demonstrated that PGE_2_ selectively inhibited NLRP3 inflammasome activation through E-prostanoid 4 receptor-mediated induction of cyclic AMP pathway.^46, 47^ We show here that hUCB-MSCs can remarkably suppress NLRP3 inflammasome-mediated IL-1*β* production in macrophages through enhanced COX-2 signaling in response to IL-1*β*. Given that PGE_2_ is the main product of COX-2 signaling and COX-2/PGE_2_ pathway is one of the pivotal signals responsible for the immunosuppressive effects of MSCs, our results suggest that direct inhibition of NLRP3 inflammasome can be one of the core strategies of hUCB-MSCs immunomodulation to attenuate excessive inflammatory responses, particularly in RA microenvironment. Although NO was previously proposed to be a negative regulator of the NLRP3 inflammasome in human and mouse,^48^ we observed that blocking NO synthesis in hUCB-MSCs failed to affect the suppressive effects on the NLRP3 inflammasome. In our own previous studies, we showed that the level of NO production by hUCB-MSCs was very low and that the inhibition of NO synthesis did not alter the modulatory effect of hUCB-MSCs on various types of immune cells, consistently indicating that NO may be a redundant factor at least in hUCB-MSC-mediated suppression of NLRP3 inflammasome activation.^17^ Further investigations are required to identify the principal mediators and clarify the interplay between the NLRP3 inflammasome and MSCs.

It is well known that the anti-inflammatory effects of MSCs are mediated by the production of soluble factors such as PGE_2_, IDO, NO, transforming growth factor-beta and hepatocyte growth factor.^15, 16, 29, 30^ Recently, we showed that hUCB-MSCs and hBM-MSCs exhibited different level of basal PGE_2_ secretion with divergent pattern of responses to inflammatory stimuli,^10^ indicating that the immunomodulatory potency and mechanisms of MSCs can differ depending on the source of the MSCs. Moreover, several studies from other groups have demonstrated that the immunomodulatory properties of MSCs could be induced by the inflammatory cytokines secreted by activated immune cells.^29, 31^ Therefore, the disease-specific inflammatory milieu can be critical to the therapeutic effects of MSCs. In this study, we found that the ability of hUCB-MSCs to induce the M2 phenotype is mediated by the concerted action of COX-2 and TSG-6 signaling, which is enhanced by TNF-*α*. hUCB-MSCs also remarkably reduced the NLRP3 inflammasome-mediated IL-1*β* production in macrophages, a result that may be mediated by the enhanced COX-2 signaling in response to IL-1*β*. These findings suggest that injected MSCs can effectively respond to the RA-specific immune microenvironment to reduce the arthritic inflammatory responses and subsequent clinical manifestations.

Current extensive investigations into RA have led to the development of targeted therapies that block specific cytokine networks or cellular pathways. Among these therapies, anti-TNF-*α* biologics represent the front line of treatment for active RA.^4^ However, the efficacy of these treatments is still limited, and there is uncertainty about long-term safety.^5, 6, 38^ We revealed here that hUCB-MSCs can remarkably suppress NLRP3 inflammasome-mediated IL-1*β* production, as well as TNF-*α* secretion. Although the current biologic therapies target a single cytokine, hUCB-MSCs can simultaneously regulate multiple macrophage-derived cytokines and contribute to the re-establishment of systemic immune balance. Therefore, cell therapy using hUCB-MSCs can be successfully used in patients who do not respond to current biologic therapies.

Herein, our findings are novel in that we further proved this mode of action using immune cells obtained from RA patients. In this study, patients with RA generally showed up-regulated cytokine profiles, including TNF-*α*, IL-1*β*, IL-6 and IL-10, compared with healthy individuals. Surprisingly, co-culture with hUCB-MSCs significantly reduced the level of inflammatory cytokines produced by those PBMCs exhibiting relatively higher production of these cytokines, whereas this cytokine-regulating effect was not observed when hUCB-MSCs were co-cultured with PBMCs showing low levels of cytokine secretion (data not shown). These findings consistently support the hypothesis that inflammatory conditions stimulate the immunosuppressive effects of hUCB-MSCs and more importantly suggest that hUCB-MSCs are responsive to immunologic environment in disease. Furthermore, hUCB-MSCs efficiently polarized patient macrophages toward the M2 phenotype to a greater extent than primary cultured or THP-1-derived macrophages. Although many studies have demonstrated the anti-inflammatory mechanisms of MSCs on immune cells, only a few groups have verified these effects both *in vitro* and *ex vivo* using disease-specific target cells. Taken together, these findings suggest that hUCB-MSC-mediated macrophage modulation has therapeutic potential in patients with RA.

In conclusion, our study revealed that hUCB-MSCs can act as cellular modulators of macrophages by simultaneously regulating the production of TNF-*α* and IL-1*β* and that the systemic administration of hUCB-MSCs could be an attractive therapeutic alternative for RA.

## Materials and methods

### Isolation and culture of hUCB-MSCs

Fresh hUCB samples were obtained from the Seoul City Boramae Medical Center Cord Blood Bank after full-term delivery with written consent of the mother. This study was approved by the Institutional Review Board (IRB) of Boramae Medical Center and Seoul National University (IRB no. 0603/001-002-07C1). hUCB-MSCs were prepared and verified as described previously.^49^

### Mice

6- to 8-week-old male DBA1/J mice were purchased from Jackson Laboratory (Bar Harbor, ME, USA) and maintained in specific pathogen-free facility of the Seoul National University. All mice were stabilized for at least 1 week and then immunized with bovine type II collagen to induce arthritic symptoms. All *in vivo* experimental procedures were approved by the Institute of Laboratory Animal Resources of Seoul National University (approval no. SNU-151203-2) and completed in compliance with the approved guidelines.

### CIA induction and treatment

RA-like symptoms were induced by the intradermal injection of bovine type II collagen (CII, Chondrex Inc., Redmond, WA, USA) into DBA1/J mice as previously described.^50^ Briefly, 100 *μ*g of CII was emulsified in an equal volume of complete Freund's adjuvant (CFA, Sigma-Aldrich, St. Louis, MO, USA). Mice received a primary immunization with the CII emulsion at the base of the tail, followed by a boosting immunization on day 21 using the same preparation and injection methods. The clinical severity of CIA was assessed by a blinded monitor every 2–3 days using the macroscopic scoring system as established previously.^51^

To evaluate therapeutic efficacy, treatment was started after the onset of disease when the arthritis score reached 3 or more. Mice with established CIA received daily intraperitoneal (i.p.) injections of 10^6^ hUCB-MSCs or 100 *μ*g of etanercept as a drug control for 5 days (Figure 1a). Human dermal FBs were injected as a cell control. Alternatively, other mice were given a single intravenous (i.v.) injection of 10^6^ hUCB-MSCs at the same phase of disease progression (Figure 2a). After sacrifice on day 49, blood and major organs were collected for cytokine analysis and histopathological evaluation.

### Histopathologic evaluation

Formalin-fixed limb samples were decalcified with 10% formic acid. Decalcified limbs were subsequently processed and embedded in paraffin according to standard histologic procedures. Serial 5-*μ*m-thick sections were prepared, stained with hematoxylin and eosin (H&E) and then assessed microscopically for the extent of inflammation and joint destruction according to previously reported guidelines.^52^ Sections were stained with safranin O or toluidine blue for precise evaluation of cartilage destruction.

### Generation and stimulation of macrophages

hUCB-derived primary macrophages were isolated and differentiated as previously described.^53^ Briefly, MNCs were first isolated from hUCB, followed by the separation of CD14^+^ monocytes using CD14 microbeads (Miltenyi Biotech, Bergisch Gladbach, Germany). To generate macrophages, monocytes were incubated in six-well cell culture plates at a density of 10^5^ cells/cm^2^ for 7 days. The medium was replaced every 3 days, and the adherent cells were cultured in fresh macrophage induction medium (Supplementary Figure S2). On day 7, the adherent cells were stimulated with 1 *μ*g/ml LPS (Invivogen, San Diego, CA, USA) plus 20 ng/ml interferon gamma (IFN-*γ*, PeproTech, Rocky Hill, NJ, USA) for 48 h for the activation of macrophages.

To stimulate differentiation to macrophage-like cells, THP-1 cells were seeded in six-well plates at a density of 10^5^ cells/cm^2^ and were treated with 200 nM phorbol 12-myristate 13-acetate (PMA, Sigma-Aldrich) for 48 h, followed by stabilization with fresh RPMI 1640 medium (Gibco BRL, Grand Island, NY, USA) without PMA for 5 additional days (see Supplementary Figure S2). On day 5, fully differentiated macrophage-like cells were treated with LPS and IFN-*γ* for 48 h for classical M1 activation. Alternatively, macrophages were primed with 1 *μ*g/ml of LPS for 4 h, followed by stimulation with 5 *μ*M of nigericin (Invivogen) for 45 min for activation of the NLRP3 inflammasome.

### Flow cytometric analysis

Flow cytometry was completed with a FACS Calibur and analyzed using Cell Quest software (BD Biosciences, San Jose, CA, USA). For characterization of the macrophages obtained from hUCB and THP-1 cells, fully differentiated macrophages were harvested and resuspended in phosphate buffered saline (PBS). Macrophages were pretreated with Fc receptor blocking reagent (Miltenyi Biotech) for 10 min, followed by staining with fluorophore-conjugated antibodies specific for CD3, CD4, CD11b, CD14, CD44, CD73, CD105, CD206 and HLA-DR or the respective isotype controls (BD Biosciences) for 1 h. To assess M2 polarization, resting macrophages were detached after co-culture with hUCB-MSCs in direct or transwell conditions for 48 h. Subsequently, cells were stained with FITC-conjugated anti-CD14 and either PE-conjugated anti-CD206 or PE-conjugated anti-CD36 (BD Biosciences).

### Cytokine profile

Classically activated macrophages were co-cultured with hUCB-MSCs for 48 h, after which the culture supernatant was harvested. The secretion of TNF-*α* and IL-10 from macrophages was measured using a commercial enzyme-linked immunosorbent assay (ELISA) kit (R&D Systems, Minneapolis, MN, USA). The production of IL-1*β* and active caspase-1 in response to NLRP3 stimulation was quantified using an ELISA kit (R&D Systems).

### RNA interference

For selective knockdown of TSG-6, hUCB-MSCs were transfected with 25 nmol/l of siRNA for TSG-6 (Santa Cruz Biotechnology, Dallas, TX, USA) or scrambled siRNA control (GE Dharmacon, Lafayette, CO, USA) using Lipofectamine RNAiMAX (Invitrogen, Carlsbad, CA, USA) when confluency of the cells reached around 60% according to the manufacturer's recommendation. To confirm successful knockdown of TSG-6 expression, RNA and protein were extracted at 48 h after the transfection and analyzed by RT-PCR and western blotting.

### Patient samples and *ex vivo* experiments

Peripheral blood and serum samples were obtained from either patients diagnosed with RA or healthy individuals. RA was diagnosed according to the 2010 American College of Rheumatology-European League Against Rheumatism (ACR-EULAR) classification criteria; fundamental patient information and clinical characteristics are shown in Table 1. All the protocols utilizing patient samples were approved by the IRB of Boramae Medical Center (no. 20131226/16-2013-175-011), and all participants gave written informed consent. PBMCs were isolated and maintained *ex vivo* as previously described.^23^

### Western blot

Cells were detached and lysed in Pro-prep solution (iNtRon Biotechnology, Seongnam, Korea) supplemented with protease inhibitors. Extracted protein lysates were quantified by Bradford method using Bio-Rad protein assay (Bio-Rad Laboratories, Hercules, CA, USA) and 12 *μ*g of protein was loaded to 12% or 15% of sodium dodecyl sulfate-polyacrylamide gel electrophoresis, followed by transferred to nitrocellulose membranes (Bio-Rad Laboratories). Membranes were blocked with Tris-buffered saline containing 3% bovine serum albumin (Sigma-Aldrich) and incubated with primary antibodies, including COX-2 (Abcam, Cambridge, UK), TSG-6 (R&D Systems), *α*-tubulin and glyceraldehyde-3-phosphatate dehydrogenase (Millipore, Billerica, MA, USA). After incubation with horseradish peroxidase-conjugated secondary antibodies, the extent of protein expression was detected by enhanced chemiluminescence reagent (GE Healthcare Life Science, Buckinghamshire, UK).

### Statistical analysis

All data are presented as the mean±S.D. Variables were analyzed with Student's *t*-tests for comparisons between two conditions or one-way ANOVAs followed by the Bonferroni *post hoc* test for comparisons of more than two groups. Two-way ANOVAs were performed for comparisons of multiple time points. All statistical analysis were conducted using GraphPad Prism version 5.01 (GraphPad Software, San Diego, CA, USA), and *P*-values <0.05 were defined as statistically significant. The level of statistical significance indicated by the asterisks is provided in the figure legends.

## Figures and Tables

**Table 1:** Fundamental profile of patients with RA enrolled in this study^a^

	RA patients (*n*=15)
*Demographics*	
Age, years	56.1±12.3
Gender, % female	60
	
*Clinical characteristics*	
RF and/or anti-CCP antibody, % positive	86.7
Disease duration, years	7.0±11.6
Patients global assessment of disease activity score, 0–10	4±2.5
Routine assessment of patient index data with 3 measurement score	9.1±5.5
ESR, mm/h	46.3±33.3
CRP, mg/dl	1.7±1.4
	
*Prescription history*	
Naive to treatment, %	26.7
PDS, %	66.7
PDS dose, mg/week	4.8±1.8
MTX, %	60
MTX dose, mg/week	13.9±1.7
Combined PDS with MTX, %	53.3

Abbreviations: CCP, citrullinated protein; CRP, C-reactive protein; ESR, erythrocyte sedimentation rate; MTX, methotrexate; PDS, prednisolone; RF, rheumatoid factor

a Values are presented as the mean±S.D.
