# Supplementary material for: Human umbilical cord blood-stem cells direct macrophage polarization and block inflammasome activation to alleviate rheumatoid arthritis
Source: Cell Death Dis. 2016 Dec 22;7(12):e2524–. doi: 10.1038/cddis.2016.442 (PMC5260999; doi:10.1038/cddis.2016.442)
Supplement: Supplementary Information [file cddis2016442x1.pdf]

Supplementary Information

Human Umbilical Cord Blood-Stem Cells Direct Macrophage Polarization and Block Inflammasome Activation to Alleviate Rheumatoid Arthritis

Tae-Hoon Shin, Hyung-Sik Kim, Tae-Wook Kang, Byung-Chul Lee, Hwa-Yong Lee, Yoon-Jin Kim, Ji-Hee Shin, Yoojin Seo, Soon Won Choi, Seunghee Lee, Kichul Shin, Kwang-Won Seo, and Kyung-Sun Kang*

Running title: Regulation of macrophage function by MSCs in RA

Supplemental Figure Legends

Figure S1. Intravenously injected hUCB-MSCs are primarily distributed in the lung and joints and are excreted within one week.

(A-D) Arthritis was induced by immunizing mice with bovine type II collagen (CII) mixture with complete Freund's adjuvant (CFA). hUCB-MSCs were infused intravenously (i.v.), and mice were sacrificed 2 hours (A), 3 days (B), 1 week (C) and 2 weeks (D) after cell injection. At the same time, DNA was obtained from the major organs. The concentration of xenogeneic hUCB-MSCs in mice with collagen-induced arthritis (CIA) was evaluated using real-time qPCR with the human-specific ALU gene. **(E-F)** Changes in the distribution of injected cells throughout the joint (E) and lung tissue (F) were analyzed over time. 3 mice were included in each time point, and the detection limit was determined on the basis of the standard curve (0.003% of the human ALU gene).

Figure S2. Differentiation and characterization of human umbilical cord blood- and THP-1 cell-derived macrophages.

(A) Similarities between human umbilical cord blood- (hUCB-) and THP-1 cell-derived macrophages were determined using the following protocols. **(B)** Representative phase-contrast images of fully differentiated macrophages, scale bar = 100 µm. **(C)** The expression of CD68, a typical macrophage lineage marker, was confirmed with immunocytochemistry (ICC), scale bar = 50 µm. Data are representative of three independent experiments. **(D)** Identification of various immune cell populations in hUCB-derived mononuclear cells and the purity of CD14-sorted monocyte/macrophage populations from two different donors was evaluated with flow cytometry. Data in table are presented as the mean ± SD from the cumulative results of at least three independent experiments. **(E-F)** The relative proportion of

each immune cell population, including lymphocytes, granulocytes and monocytes, was determined with flow cytometry with specific lineage markers. The results are shown as the mean ± SD from three independent experiments. **(G)** Representative surface marker expression on fully differentiated hUCB- and THP-1-derived macrophages was analyzed with flow cytometry. One representative of at least three independent experiments is shown.

Figure S3. Enhanced COX-2 and/or TSG-6 signaling in hUCB-MSCs upon TNF-α and IL-1β stimulation contributes to macrophage regulation.

(A) The expression of CD206 by $CD14^+$ macrophages after co-culture with hUCB-MSCs in direct contact or transwell conditions was determined with flow cytometry. **(B)** hUCB-MSCs obtained from two different donors were cultured with or without recombinant human TNF-α for 2 days, after which the protein levels of the pivotal factors COX-2 and TSG-6 were quantified with western blotting. **(C-D)** The expression of these factors was altered after pre-incubation with conditioned medium from macrophages (C) or pre-treatment with IL-1β (D). One representative experiment of three or the cumulative of at least three independent experiments are shown. The results are shown as the mean ± SD. *** $P<0.001$ (one-way ANOVA followed by the Bonferroni post hoc test).

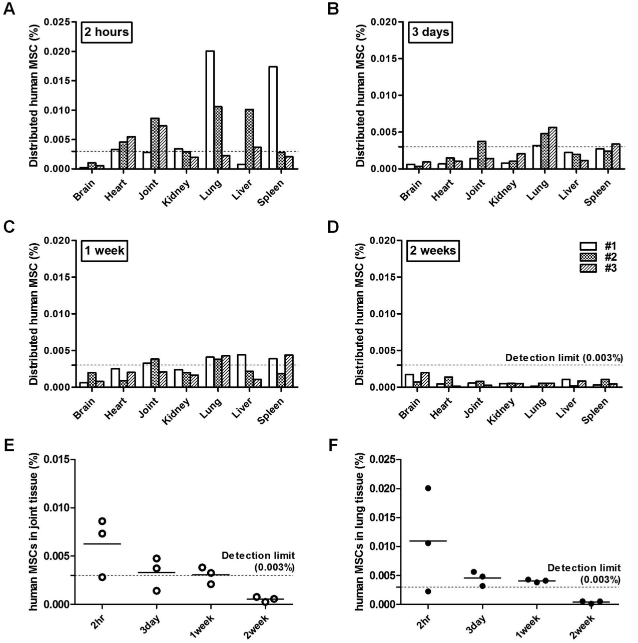

Kyung-Sun Kang, Figure S1

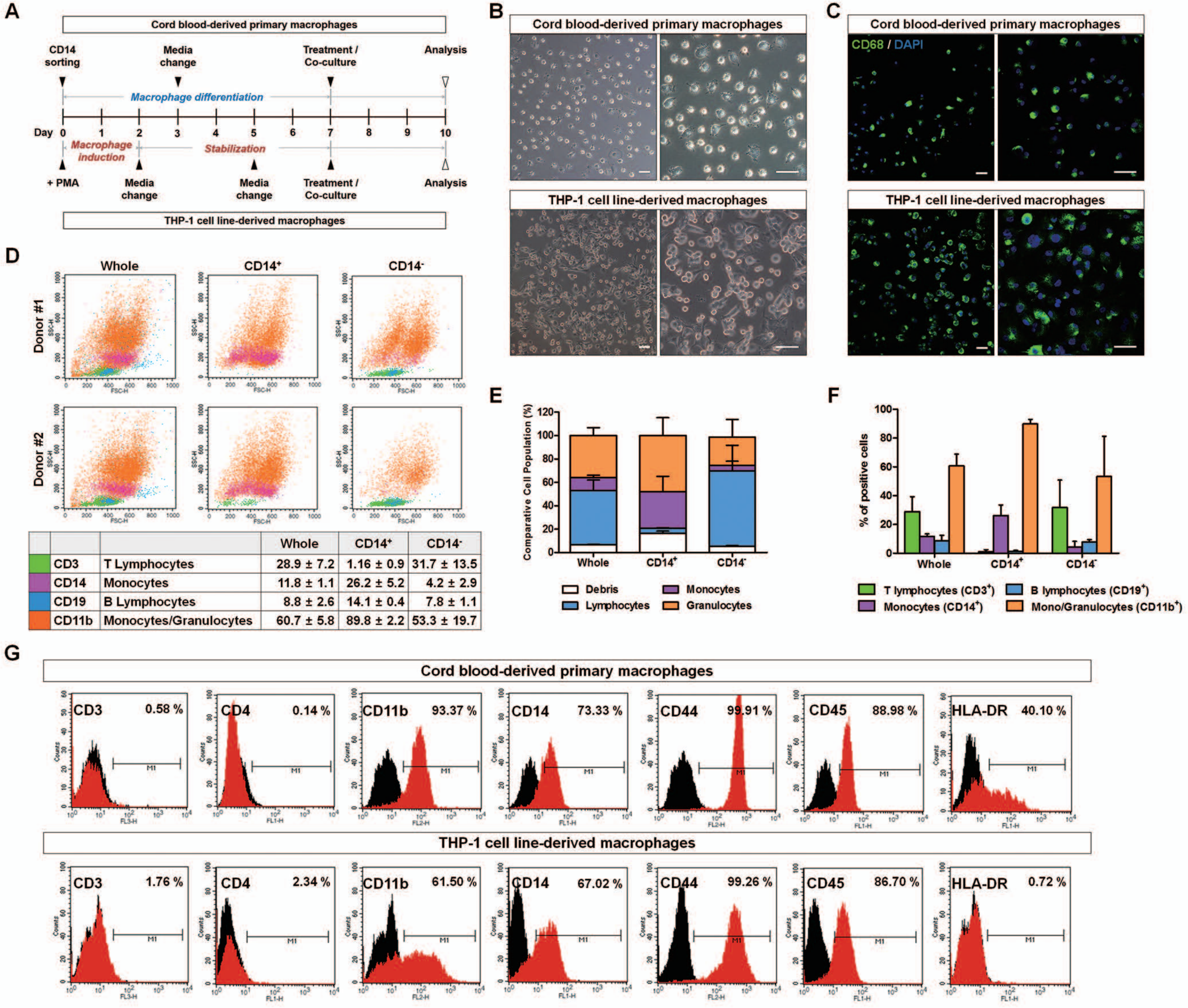

			Whole	CD14+	CD14-
	CD3	T Lymphocytes	28.9 ± 7.2	1.16 ± 0.9	31.7 ± 13.5
	CD14	Monocytes	11.8 ± 1.1	26.2 ± 5.2	4.2 ± 2.9
	CD19	B Lymphocytes	8.8 ± 2.6	14.1 ± 0.4	7.8 ± 1.1
	CD11b	Monocytes/Granulocytes	60.7 ± 5.8	89.8 ± 2.2	53.3 ± 19.7

Kyung-Sun Kang, Figure S2

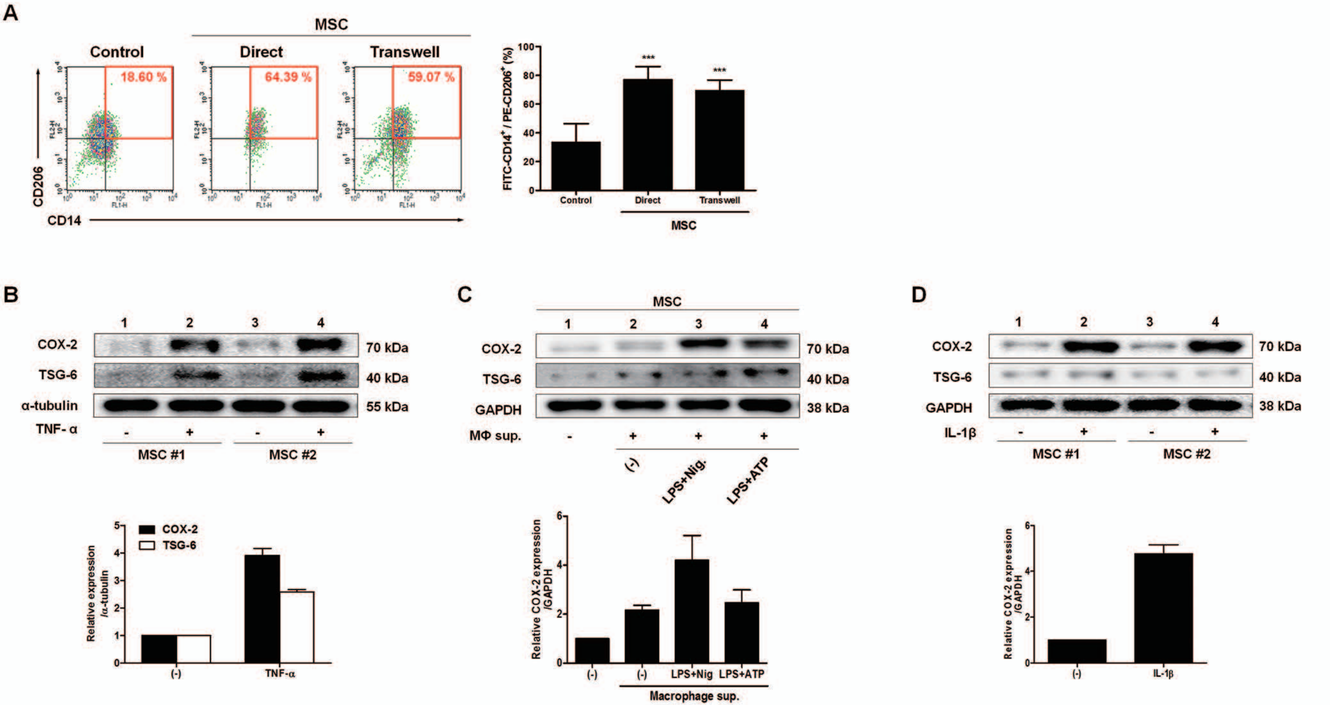

Kyung-Sun Kang, Figure S3
